# Physiologic and laboratory correlates of depression, anxiety, and poor sleep in liver cirrhosis

**DOI:** 10.1186/1471-230X-13-18

**Published:** 2013-01-22

**Authors:** Fang-Yuan Ko, Albert C Yang, Shih-Jen Tsai, Yang Zhou, Lie-Ming Xu

**Affiliations:** 1 Section of Liver Cirrhosis, Shuguang Hospital and Institute of Liver Diseases, Shanghai University of Traditional Chinese Medicine, 528 Zhangheng Road, Shanghai, 201203, China; 2 Department of Psychiatry, Taipei Veterans General Hospital, Taipei, Taiwan; 3 Division of Psychiatry, School of Medicine, National Yang-Ming University, Taipei, Taiwan; 4 Center for Dynamical Biomarkers and Translational Medicine, National Central University, Chungli, Taiwan

**Keywords:** Liver cirrhosis, Psychological distress, Heart rate variability

## Abstract

**Background:**

Studies have shown psychological distress in patients with cirrhosis, yet no studies have evaluated the laboratory and physiologic correlates of psychological symptoms in cirrhosis. This study therefore measured both biochemistry data and heart rate variability (HRV) analyses, and aimed to identify the physiologic correlates of depression, anxiety, and poor sleep in cirrhosis.

**Methods:**

A total of 125 patients with cirrhosis and 55 healthy subjects were recruited. Each subject was assessed through routine biochemistry, 5-minutes ECG monitoring, and psychological ratings of depression, anxiety, and sleep. HRV analysis were used to evaluate autonomic functions. The relationship between depression, sleep, and physiologic correlates was assessed using a multiple regression analysis and stepwise method, controlling for age, duration of illness, and severity of cirrhosis.

**Results:**

Reduced vagal-related HRV was found in patients with severe liver cirrhosis. Severity of cirrhosis measured by the Child-Pugh score was not correlated with depression or anxiety, and only had a weak correlation with poor sleep. The psychological distress in cirrhosis such as depression, anxiety, and insomnia were correlated specifically to increased levels of aspartate aminotransferase (AST), increased ratios of low frequency to high frequency power, or reduced nonlinear properties of HRV (α_1_ exponent of detrended fluctuation analysis).

**Conclusions:**

Increased serum AST and abnormal autonomic nervous activities by HRV analysis were associated with psychological distress in cirrhosis. Because AST is an important mediator of inflammatory process, further research is needed to delineate the role of inflammation in the cirrhosis comorbid with depression.

## Background

Cirrhosis is a consequence of chronic liver disease commonly caused by alcoholism, viral hepatitis, autoimmune disorders, or other aetiologies. The prevalence of liver cirrhosis is high in most Asian countries where chronic hepatitis B or C are common [1]. Cirrhosis places a significant burden of psychological stress on affected individuals [2,3]. In patients with liver cirrhosis, quality of life is considerably impaired and associated with mild to moderate depression and anxiety [4-6]. In a study of one hundred and fifty-six Italian patients with liver cirrhosis, Bianchi et al. [6] demonstrated that the global score of Psychological General Well-Being Index was severely reduced compared to the population norm and the score was significantly correlated with the severity of liver failure as assessed by the Child–Pugh (CP) score.

Despite the relationship between cirrhosis and distressed psychological states being well-documented in aforementioned studies, no study has evaluated the laboratory and physiologic correlates of depression, anxiety, or poor sleep commonly seen in patients with cirrhosis. Furthermore, these psychological manifestations are known risk factors for cardiovascular morbidity and are also associated with autonomic dysregulation. A body of research has emerged demonstrating that reduced vagal modulation or increased sympathetic activity are associated with anxiety [7-9], depression [10], and insomnia [11-13]; hence, the physiological and psychological stress of cirrhosis may be measured by heart rate variability (HRV), a non-invasive measure of the cardiac autonomic nervous system.

Functional limitations, altered cardiac autonomic activity, and psychological distress are known disorders in patients with liver cirrhosis, relating to increased morbidity and mortality. However, the inter-relationship between these morbidity remains unclear. The aim of this study was therefore to examine the influence of severity of cirrhosis on emotional parameters and heart rate variability (HRV) indices, as well as to determine whether emotional distress contributes to autonomic dysfunction in these patients. To this end, this study measured both the biochemical data and HRV indices, and identified the physiologic and laboratory correlates of depression, anxiety, and poor sleep in patients with liver cirrhosis.

## Methods

### Patients

The study sample consisted of 125 patients with liver cirrhosis (73 males, 52 females; mean age = 58.3 ± 11.6 years) and 55 healthy controls (29 males, 26 females; mean age = 58.0 ± 11.8 years) recruited from the outpatient clinics of the Section of Liver Cirrhosis, Shuguang Hospital, China. Inclusion criteria were a positive diagnosis of liver cirrhosis, documented by histology through a liver biopsy, or through clinical, sonographic and endoscopic evidence of portal hypertension and confirmed with laboratory data. Exclusion criteria were (1) a history of major mental illness such as schizophrenia, major depression, or bipolar disorder; (2) current use of psychotropic medication; (3) active infections; (4) hepatocellular carcinoma evidenced by sonographic focal liver lesion or α-fetoprotein exceeding ten times the upper limits of normal values. Of note: patients with viral hepatitis were neither actively treated with interferon at the time of investigation or during the previous year. The control subjects had neither history of mental illness nor liver diseases.

Demographic and clinical variables included age, gender, duration of illness, history of viral hepatitis and alcohol dependence, routine biochemistry, and the five items of the CP score (albumin and total bilirubin levels, prothrombin activity, presence and severity of ascites and encephalopathy) [14]. Of 125 patients, 43 were classified as CP class A, 58 were class B, and 24 were class C. The study protocol was in accordance with the guidelines for clinical research and was approved by the Institutional Review Board and the Ethical Review Committee of the hospital. Informed consent was obtained after all subjects had been fully informed of its purpose.

### Psychological measurements

Depression severity was evaluated with the Hamilton Depression Rating Scale (HAMD, 24 items) [15], and anxiety was evaluated with the Hamilton Anxiety Scale (HAMA) [16]. Subjective sleep quality was assessed through the Pittsburgh Sleep Quality Index (PSQI). The ratings were administered by experienced clinical raters certified with high rates of inter-rater reliability and levels of procedural integrity. The self-reported depression scale, such as Beck Depression Inventory (BDI), [17] was not used in this study because the Chinese version of BDI was found to have low reliability in the Chinese population due to cultural bias on depression [18]. The objective rating of depression by HAMD and HAMA may be more appropriate than self-reported measure in our study population.

### ECG monitoring

A customized ECG recording device was used to obtain a 5-minute ECG from each recruited subject [19]. ECG signals were recorded at a sampling rate of 256 Hz, and were automatically processed and analyzed by open source HRV algorithms [20]. All ECG monitoring took place during the daytime, and participants were asked to avoid smoking and to remain in a resting state while being monitored.

### Analysis of heart rate variability

The standard HRV analysis has been well reviewed [21]. Time domain measures of HRV include the mean heart rate and standard deviation of the normal interbeat intervals (SDNN), and the root mean square successive difference between adjacent normal interbeat intervals (RMSSD). The SDNN assesses the overall variability of interbeat intervals. The RMSSD measures the short-term variation of interbeat intervals, which is primarily modulated by parasympathetic innervation [22]. Standard spectral HRV measures [21] include high-frequency power (HF; 0.15–0.40 Hz), low-frequency power (LF; 0.04–0.15 Hz), and very low-frequency power (VLF; 0.003–0.04 Hz). LF power is suggested to be modulated by both sympathetic and parasympathetic activities, whereas HF power is mainly modulated by parasympathetic activity [23,24]. The LF/HF ratio is considered a measure of the shift of sympathovagal balance toward sympathetic activity [21,25]. The physiological mechanism underlying VLF power is disputed but has been suggested to be mediated partly by the renin–angiotensin–aldosterone system or by parasympathetic modulation [26].

In addition, we incorporated a nonlinear HRV index: a Detrended Fluctuation Analysis (DFA) [27,28]. DFA quantifies the presence of long-range (fractal) correlations inherent in physiologic signals and is therefore a complexity measure. The details of the DFA method is available at Physionet (http://physionet.org), a research resource for complex physiologic signals [20]. The root-mean-square fluctuation of integrated and detrended time series was measured at different observation windows and plotted against the size of the observation window on a log-log scale. The scaling exponent α is then derived from the slope of line fitting to the generated log-log plot. The short-term exponent α_1_ (4 to 11 heartbeats) and the long-term scaling exponents α_2_ (>11 heartbeats) were also calculated [27,29]. Low-exponent values represent reduced fractal properties of heart rate dynamics and have been implicated in increased risks of fatal cardiac arrhythmia, increased mortality, or poor prognosis in cardiovascular diseases [30-33].

### Statistical analysis

Statistical Package for the Social Sciences (SPSS version 15.0, Chicago, IL) software was used for the statistical analyses. The spectral HRV indices were log transformed to produce normalized distributions. Chi-squared tests were used to compare categorical variables. One-way Analysis Of Variance (ANOVA) was used to test for differences in demographical, clinical, and HRV measures among groups according to CP class, and post hoc Bonferroni tests were used for paired-group comparisons. Partial correlation controlling for age and duration of illness was used to test the association between CP scores and psychological distress as well as HRV indices. The relationship between depression, sleep, and physiologic correlates was assessed with multiple regression analyses using the backward selection method, controlling for age, duration of illness, and CP score. The physiologic correlates included biochemical data and HRV indices. We estimated the total sample size by the power analysis to be at least 118 by assuming power of 80%, 5% significance level, and a total of 10 predictors. The Variance Inflation Factor (VIF) was estimated for all physiologic correlates and a VIF value of 10 or greater was considered an indication of significant co-linearity. A p value of less than 0.05 (two-tailed) was required for all statistical comparisons.

## Results

### Patients

Table 1 shows the profiles of demographic, clinical and psychological ratings, as well as HRV data according to CP class. Three groups did not differ in age, gender and duration of illness. Regarding the aetiologies of liver cirrhosis, patients with CP class B and C had higher rate of alcoholism, compared to those with CP class A. The ANOVA test showed significant between-group differences in serum levels of albumin (p < 0.001), aspartate aminotransferase (AST; p < 0.001), total bilirubin (p < 0.001), and direct bilirubin (p < 0.001). As expected, post-hoc tests revealed that assessments of biochemistry were in accordance with the CP class.

**Table 1:** Demographic, clinical, psychological, and heart rate variability characteristics

**Characteristics**	**Healthy control** **(N = 55)**	**Child-Pugh class**			***p***	**Post-hoc**
		**A (N = 43)**	**B (N = 58)**	**C (N = 24)**		
**Demographic**						
Age, year	58.0 ± 11.8	56.6 ± 12.0	58.6 ± 11.1	60.7 ± 12.1	0.578	
Gender, Male (%)	29 (52.7)	25 (58.1)	36 (62.1)	12 (50.0)	0.680	
**Clinical**						
Child-Pugh score		5.6 ± 0.5	7.9 ± 0.8	10.5 ± 0.9	**<0.001**	A < B < C
Duration of illness, year		3.2 ± 3.4	3.1 ± 4.0	3.5 ± 3.6	0.940	
Viral hepatitis, N (%)		32 (74.4)	37 (63.8)	14 (58.3)	0.346	
Alcoholism, N (%)		1 (2.3)	8 (13.8)	5 (20.8)	**0.048**	A < B < C
Parasite, N (%)		2 (4.7)	1 (1.7)	2 (8.3)	0.368	
Autoimmune, N (%)		2 (4.7)	3 (5.2)	0 (0)	0.533	
Hepatic-encephalopathy, N (%)		0 (0)	5 (8.6)	2 (8.4)	0.143	
Ascites, N (%)		3 (7.0)	21 (36.2)	21 (87.5)	**<0.001**	A < B < C
Albumin, g/L		35.4 ± 4.5	26.8 ± 4.9	26.2 ± 4.7	**<0.001**	A > B = C
ALT, U/L		46.7 ± 44.7	39.3 ± 34.1	59.7 ± 77.7	0.233	
AST, U/L		40.2 ± 21.7	57.5 ± 34.5	79.9 ± 54.2	**<0.001**	A = B < C
GGT, U/L		71.2 ± 87.0	68.0 ± 69.4	95.8 ± 105.6	0.384	
ALP, U/L		102.4 ± 60.6	114.2 ± 56.7	135.0 ± 59.9	0.105	
T-Bil, μmol/L		23.2 ± 7.4	42.3 ± 38.8	91.9 ± 47.1	**<0.001**	A < B < C
D-Bil, μmol/L		6.4 ± 3.2	14.1 ± 21.3	44.3 ± 33.9	**<0.001**	A = B < C
INR		1.24 ± 0.14	1.66 ± 1.68	1.69 ± 0.36	0.185	
**Psychological measurements**						
PSQI		6.4 ± 3.2	7.6 ± 4.6	10.0 ± 5.2	**0.007**	A = B < C
HAMD		10.0 ± 10.4	11.2 ± 10.2	17.4 ± 9.6	**0.017**	A = B < C
HAMA		5.8 ± 4.9	6.6 ± 5.9	8.8 ± 6.2	0.125	
**Heart rate variability**						
Mean heart rate, beats/minute	84.4 ± 11.8	75.4 ± 11.0	80.1 ± 14.0	83.8 ± 16.7	**0.011**	H > A
SDNN, ms	42.9 ± 15.1	34.7 ± 15.3	33.3 ± 20.5	22.8 ± 13.8	**<0.001**	H = A = B > C
RMSSD, ms	28.2 ± 10.1	29.7 ± 14.8	27.8 ± 19.8	18.3 ± 11.7	**0.035**	H = A = B > C
VLF power, ln(ms^2^/Hz)	7.58 ± 0.84	6.95 ± 0.96	6.60 ± 1.37	6.19 ± 1.22	0.051	
LF power, ln(ms^2^/Hz)	6.99 ± 0.84	6.33 ± 1.12	5.84 ± 1.19	5.21 ± 1.48	**<0.001**	H = A > B > C
HF power, ln(ms^2^/Hz)	6.56 ± 0.88	6.22 ± 1.17	5.81 ± 1.26	4.91 ± 1.60	**0.044**	H = A = B > C
LF/HF ratio	1.67 ± 0.65	1.39 ± 0.98	1.29 ± 0.87	1.76 ± 1.42	0.086	
DFA, α	0.80 ± 0.10	0.77 ± 0.15	0.77 ± 0.17	0.83 ± 0.13	0.340	
DFA, α_1_	1.14 ± 0.18	0.91 ± 0.27	0.81 ± 0.31	0.89 ± 0.25	**<0.001**	H > A = B = C
DFA, α_2_	0.94 ± 0.15	0.94 ± 0.22	0.98 ± 0.23	1.02 ± 0.20	0.384	

Post-hoc: (H) healthy control; (A) Child-Pugh Class A; (B) Child-Pugh Class B; (C) Child-Pugh Class C; ALT: Alanine aminotransferase; AST: Aspartate aminotransferase; GGT: Gamma-Glutamyltransferase; ALP: Alkaline phosphatase; T-Bil: Total bilirubin; D-Bil: Direct bilirubin; INR: International normalized ratio of blood coagulation; PSQI: Pittsburgh sleep quality index; HAMD: Hamilton rating scale for depression; HAMA: Hamilton rating scale for anxiety; DFA: Detrended fluctuation analysis.

### Psychological ratings

Regarding psychological ratings (Table 1), significant between-group differences were found in PSQI (p = 0.007), and HAMD (p = 0.017). Patients with CP class C had significantly higher PSQI and HAMD ratings than patients with CP class A or B, suggesting poorer sleep and more severe depressed moods in cases of advanced liver cirrhosis.

### HRV analysis

For HRV measures (Table 1), significant between-group differences were found in mean heart rate (p = 0.011), SDNN (p < 0.001), RMSSD (p = 0.035), LF power (p < 0.001), HF power (p = 0.044), and DFA α_1_ (p < 0.001). Patients with CP class C had a significantly decreased SDNN and LF, and decreased vagal-related HRV such as RMSSD and HF power, compared to controls and those with CP class A or B.

### Correlation between CP score and psychological ratings / HRV analysis

Partial correlation analysis controlling for age and duration of illness indicated that CP scores were significantly correlated to levels of PSQI (r = 0.210; p = 0.021), and sub-components of PSQI, including subjective sleep quality (r = 0.217; p = 0.017) and sleep efficiency (r = 0.202; p = 0.027). CP scores were not correlated to HAMD and HAMA ratings. Regarding HRV indices, CP scores were correlated negatively to RMSSD (r = −0.231; p = 0.020), HF power (r = −0.345; p < 0.001), and positively to mean heart rate (r = 0.277; p = 0.005).

### Correlation between physiologic measures and psychological ratings

Table 2 shows the physiologic correlates of depression, anxiety and sleep using multiple regression models with the stepwise method. PSQI was predicted only by AST serum levels (r = 0.224; p = 0.027). HAMD was predicted by LF/HF ratios (r = 0.222; p = 0.028), AST (r = 0.170; p = 0.045), and DFA α_1_ (r = −0.186; p = 0.041). HAMA was predicted by LF/HF ratios (r = 0.190; p = 0.039) and DFA α_1_ (r = −0.187; p = 0.041).

**Table 2:** Regression model of clinical and psychological assessment using laboratory and physiologic variables as predictors

**Psychological measurement**	**Model summary**			
	**β**	**SE**	**Partial correlation**	***P***
**PSQI**				
AST	0.025	0.011	0.224	0.027
**HAMD**				
LF/HF ratio	2.777	1.247	0.222	0.028
AST	0.062	0.036	0.170	0.045
DFA, α_1_	−11.72	6.27	−0.186	0.041
**HAMA**				
LF/HF ratio	1.351	0.713	0.190	0.039
DFA, α_1_	−4.683	2.506	−0.187	0.041

PSQI: Pittsburgh sleep quality index; HAM-D: Hamilton rating scale for depression; HAM-A: Hamilton rating scale for anxiety.

## Discussion

The key findings of this study were: (1) reduced vagal-related HRV was found in patients with CP class C; (2) severity of cirrhosis as measured by CP scores was not correlated to depression or anxiety, and was only weakly correlated with poor sleep; (3) the psychological distress in cirrhosis such as depression, anxiety, and insomnia were correlated specifically to increased levels of AST, increased LF/HF ratios, or reduced nonlinear properties of HRV – DFA α_1_. These results complemented prior research and identified the physiologic factors associated with the severity of psychological distress in patients with cirrhosis.

Findings of compromised psychological status related to the severity of cirrhosis were partially consistent with prior studies [4-6,34,35]. Although patients with CP class C showed significant psychological distress in sleep (PSQI) and depression (HAMD), we only found a weak correlation between CP scores and PSQI and its sub-component. HAMD and HAMA scores, indicative of depressed moods and anxiety, are not correlated with cirrhosis severity (CP score) in our study. The aetiology of depression in patients with liver cirrhosis remains largely unknown [36]. This finding did not support the study by Bianchi et al. that found a correlation between CP scores and Beck Depression ratings [6]. By contrast, we found that both PSQI and HAMD scores were correlated with levels of AST, a more direct measure of liver damage. This finding may provide a preliminary evidence of the link between liver damage and psychological distress and suggests that individual biochemical data may be more sensitive than CP scores in reflecting the psychological burden of liver cirrhosis.

The cause of differential association between AST/ALT markers and psychological ratings remains unknown. One possible explanation is that AST/ALT ratio are commonly elevated in cirrhosis patients and is a dependent marker of fibrosis stage and cirrhosis [37]. In this study, there were about 86% of patients who had AST/ALT ratio > 1 (data not shown). The elevation of AST may be a more sensitive marker to be correlated with psychological ratings. There are limited data regarding the association between the abnormal liver function and psychological distress in liver diseases. One prior study based on hepatitis C virus infected patients found that the physical function subscale in the quality of life assessment was correlated to serum AST level [38]. AST is an important mediator of the inflammatory processes, and increasing evidence has suggested that pathophysiology of depression is closely related to the proinflammatory cytokines [39], thus our findings of the association between AST and poor sleep and depression may be related to the chronic inflammation in cirrhosis, and warrants future investigation of the association between the degree of liver inflammation (e.g., necroinflammatory scores) and psychological ratings.

Previous studies have found that reduced HRV was correlated with increased severity of cirrhosis [40-43]. This study further reveals that both the severity of cirrhosis and psychological distress are correlated with changes in HRV indices. Although the causal relationship between these factors remains unclear, CP scores only had a weak correlation with poor sleep and no correlation with depression and anxiety ratings, thus suggesting that the effects of cirrhosis and psychological distress on HRV may be independent. Studies on myocardial infarctions have suggested that depression is an independent risk factor for poor cardiovascular outcomes [44-46]. Because cirrhosis has frequent cardiovascular complications [47], cirrhosis comorbid with depression may worsen cardiovascular-related morbidity and mortality and warrants future research. Of note, HAMD and HAMA scores were correlated positively with LF/HF ratios – a marker of sympathovagal balance – and negatively with DFA α_1_ – a nonlinear marker of vagal activity [29,48]. DFA α_1_ has repeatedly demonstrated its ability in predicting cardiovascular mortality [48,49], and may be instrumental in further investigations of using nonlinear HRV as a predictor of cardiovascular complications in cirrhosis.

There are several limitations in our present study. First, as the study design is cross-sectional, we cannot directly evaluate the long-term impacts of cirrhosis and psychological distress on autonomic function, and the interpretation of results is exploratory. Second, our study did not include a healthy control group; our primary interest was to investigate physiologic correlates of psychological distress within the population of cirrhosis patients. Third, the psychiatric ratings were used as a measure of psychological distress and were not for diagnostic purposes. The incidences of depression, anxiety, and poor sleep in cirrhosis patients might be addressed through psychiatric evaluation in a larger sample.

## Conclusions

In summary, this study identifies the physiologic factors associated with psychological distress in cirrhosis patients, including increased AST and abnormal autonomic-nervous activities determined through HRV analyses. Further research is needed to evaluate the use of HRV as a non-invasive tool to assess cardiovascular outcome, and to delineate the role of inflammation in the cirrhosis comorbid with depression.

## Competing interests

The authors declare that they have no competing interests.

## Authors’ contributions

FYK designed the clinical experiment, carried out the studies and drafted the manuscript. ACY and SJT performed heart rate variability and statistical analysis, and helped to draft the manuscript. YZ and LMX designed the clinical experiment, contributed study sample, and supervised the study. All authors read and approved the final manuscript.

## Pre-publication history

The pre-publication history for this paper can be accessed here:

http://www.biomedcentral.com/1471-230X/13/18/prepub
